# Prognostic role of Wnt and Fzd gene families in acute myeloid leukaemia

**DOI:** 10.1111/jcmm.16233

**Published:** 2021-01-08

**Authors:** Yifeng Dai, Zhiheng Cheng, Doerte R. Fricke, Hongyou Zhao, Wenhui Huang, Qingfu Zhong, Pei Zhu, Wenjuan Zhang, Zhihua Wu, Qing Lin, Huoyan Zhu, Yan Liu, Tingting Qian, Lin Fu, Longzhen Cui, Tiansheng Zeng

**Affiliations:** ^1^ Department of Hematology The Second Affiliated Hospital of Guangzhou Medical University Guangzhou China; ^2^ Department of Pathology and Medical Biology University of Groningen University Medical Center Groningen Groningen the Netherlands; ^3^ Translational Medicine Center State Key Laboratory of Respiratory Disease The Second Affiliated Hospital of Guangzhou Medical University Guangzhou China; ^4^ Department of Genetics LSU Health Sciences Center New Orleans LA USA; ^5^ Institute of Engineering Medicine Beijing Institute of Technology Beijing China; ^6^ Translational Medicine Center Huaihe Hospital of Henan University Kaifeng China; ^7^ Department of Hematology Huaihe Hospital of Henan University Kaifeng China; ^8^ Guangdong Provincial Education Department Key Laboratory of Nano‐Immunoregulation Tumor Microenvironment The Second Affiliated Hospital Guangzhou Medical University Guangzhou China

**Keywords:** acute myeloid leukaemia, allo‐HSCT, chemotherapy, prognosis, Wnt‐Fzd signalling pathway

## Abstract

Wnt‐Fzd signalling pathway plays a critical role in acute myeloid leukaemia (AML) progression and oncogenicity. There is no study to investigate the prognostic value of Wnt and Fzd gene families in AML. Our study screened 84 AML patients receiving chemotherapy only and 71 also undergoing allogeneic haematopoietic stem cell transplantation (allo‐HSCT) from the Cancer Genome Atlas (TCGA) database. We found that some Wnt and Fzd genes had significant positive correlations. The expression levels of Fzd gene family were independent of survival in AML patients. In the chemotherapy group, AML patients with high *Wnt2B* or *Wnt11* expression had significantly shorter event‐free survival (EFS) and overall survival (OS); high *Wnt10A* expressers had significantly longer OS than the low expressers (all *P* < .05), whereas, in the allo‐HSCT group, the expression levels of Wnt gene family were independent of survival. We further found that high expression of *Wnt10A* and *Wnt11* had independent prognostic value, and the patients with high *Wnt10A* and low *Wnt11* expression had the longest EFS and OS in the chemotherapy group. Pathway enrichment analysis showed that genes related to *Wnt10A*, *Wnt11* and *Wnt 2B* were mainly enriched in ‘cell morphogenesis involved in differentiation’, ‘haematopoietic cell lineage’, ‘platelet activation, signalling and aggregation’ and ‘mitochondrial RNA metabolic process’ signalling pathways. Our results indicate that high *Wnt2B* and *Wnt11* expression predict poor prognosis, and high *Wnt10A* expression predicts favourable prognosis in AML, but their prognostic effects could be neutralized by allo‐HSCT. Combined *Wnt10A* and *Wnt11* may be a novel prognostic marker in AML.

## 1 INTRODUCTION

The Wingless‐Int (Wnt) gene family consists of 19 structurally related genes that encode secreted lipid‐modified glycoproteins. These proteins initiate canonical and non‐canonical signalling cascades through the transmission of Frizzled (Fzd) transmembrane receptors and lipoprotein‐related protein (Lrp) single‐transmembrane co‐receptors.^1^, ^2^ Wnt signalling pathway regulates a series of cellular processes such as proliferation, differentiation, migration and apoptosis, and maintains the balance between self‐renewal and differentiation of stem cells involved in the pathogenesis of multiple types of human cancers.^3^, ^4^ In haemopoietic tissues, Wnts not only play an essential role in the maturation of haemopoietic stem cells (HSCs) into mature blood cells, but also participate in myelopoiesis by regulating erythron‐ and thrombopoiesis.^5^, ^6^ Almost all members of the Wnt and Fzd gene families are expressed at least to some extent in different haematopoietic sits during development.^7^ Therefore, Wnt signalling activity is critical in the precise regulation of normal haemopoiesis, while abnormal expression of components of Wnt‐Fzd signalling pathway can induce leukaemia.^4^

Acute myeloid leukaemia (AML) is a highly heterogeneous disease and belongs to clonal tumours originating from HSCs or myeloid cell precursors. It is associated with excessive proliferation of progenitor cells and differentiation of cell‐cycle arrest.^8^ The role of Wnt signalling in the pathogenesis of leukaemia has attracted much attention in recent years. Müller‐Tidow et al first found that the activation of Wnt signalling is a common feature of several balanced translocations in AML.^9^ A study showed that poor survival of AML patients is associated with the hypermethylation of Wnt genes without changes in expression levels.^10^ Wnt5a, as a tumour suppressor in the Wnt gene family, exhibits decreased expression in AML cells.^11^ However, Wnt4 ligand regulates leukaemic cell growth by blocking cells to G1 cell‐cycle phase in an Fzd6‐independent manner.^12^ The overexpression of *Fzd4* receptor in AML makes it highly sensitive to the activity of Wnt ligands.^13^ In addition, *Fzd3* expression is higher in AML patients compared with normal group.^14^ Therefore, the role of Wnt and Fzd gene families in the pathogenesis, development and prognosis of AML cannot be ignored.

Gene mutation and expression profiles are associated with AML progression and prognosis. Nucleophosmin 1 (*NPM1*) and fms‐like tyrosine kinase 3‐internal duplication (*FLT3‐ITD*) mutations occur in 50% and 30% of AML patients, respectively, and have negative impact on AML prognosis.^15^ Our previous studies found that individual genes in the same gene family (*FUT*, *DOK* and *PAK* families) have different prognostic value in AML.^16^, ^17^, ^18^ To investigate the prognostic significance of Wnt‐Fzd signalling pathway in AML, we analysed the effects of Wnt and Fzd gene families on survival of AML patients who either received chemotherapy alone or followed by allogeneic haematopoietic stem cell transplantation (allo‐HSCT) and dug deep into the underlying mechanism.

## 2 METHODS

### 2.1 Patients

The study screened 84 AML patients who received chemotherapy only and 71 who also underwent allo‐HSCT from the Cancer Genome Atlas (TCGA) database (https://cancergenome.nih.gov/). We obtained the complete *Wnt* (*Wnt1*, *Wnt2B*, *Wnt4*, *Wnt5B*, *Wnt6*, *Wnt8B*, *Wnt10A*, *Wnt11* and *Wnt16*) and *Fzd* (*Fzd1* to *Fzd9*) expression data in peripheral blood of 155 AML patients at diagnosis. Next, plenty of clinical and molecular characteristics were collected, including age, gender, race, white blood cell (WBC) counts, bone marrow (BM) blasts, peripheral blood (PB) blasts, French‐American‐British (FAB) subtypes and the frequencies of known recurrent genetic mutations. We also collected follow‐up data in these patients including event‐free survival (EFS) and overall survival (OS) and defined them as the end points. EFS refers to the time from diagnosis to the first event including relapse and death or was censored at the last follow‐up. OS refers to the time from diagnosis to death from any cause or the last follow‐up. Another independent cohort (GSE12417) was used to verify the results of aforesaid cohort.

### 2.2 Statistical analysis

Clinical characteristics of patients were displayed in descriptive statistics. The associations between Wnt and Fzd gene families were assessed by Spearman correlation analysis. The differences of two groups in numerical data and categorical data were calculated by Mann‐Whiney *U* test and Chi‐square test, respectively. Univariate (Kaplan‐Meier method and the log‐rank test) and multivariate (Cox proportional hazard model) analyses were used to evaluate whether Wnt and Fzd gene expression could predict EFS and OS. Spearman correlation analysis was further used to screen gene expression profiles related to Wnt genes with prognostic significance. Then we used StringDB (https://string‐db.org) for online protein‐protein interaction (PPI) analysis, used Cytoscape to visualize the results and used CytoHubba for hub gene screening. Finally, we used Metascape (http://metascape.org/) to perform pathway enrichment analysis of gene expression profiles related to Wnt genes. A two‐tailed *P* < .05 was defined as statistically significant. The SPSS 24.0 statistical software and R 3.5.0 software were used for statistical analyses, and the graphics were drawn by GraphPad Prism 7.0 software.

## 3 RESULTS

### 3.1 Association between Wnt and Fzd gene families in AML

**FIGURE 1:**
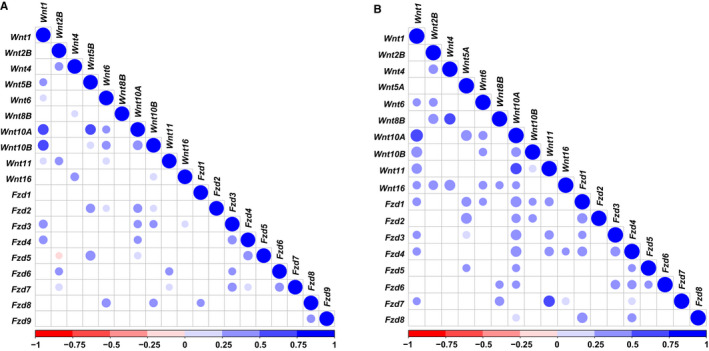
Heatmap of correlation between Wnt and Fzd gene families in patients who received chemotherapy (A) and allogeneic haematopoietic stem cell transplantation (B). Blue represents significant positive correlation, red represents significant negative correlation and a darker colour indicates a stronger correlation; blank represents no statistically significant correlation

Figure 1A shows that there were positive correlations between the expression of some genes from Wnt and Fzd families in AML patients undergoing chemotherapy only, and their correlation coefficients range from 0.215 (*Wnt11* and *Fzd7*) to 0.449 (*Wnt5B* and *Fzd5*). Figure 1B shows that there were also positive correlations between the expression of some genes from Wnt and Fzd families in AML patients receiving allo‐HSCT, and their correlation coefficients range from 0.239 (*Wnt10A* and *Fzd8*) to 0.531 (*Wnt11* and *Fzd7*).

### 3.2 Prognostic value of Wnt and Fzd gene families in AML

**FIGURE 2:**
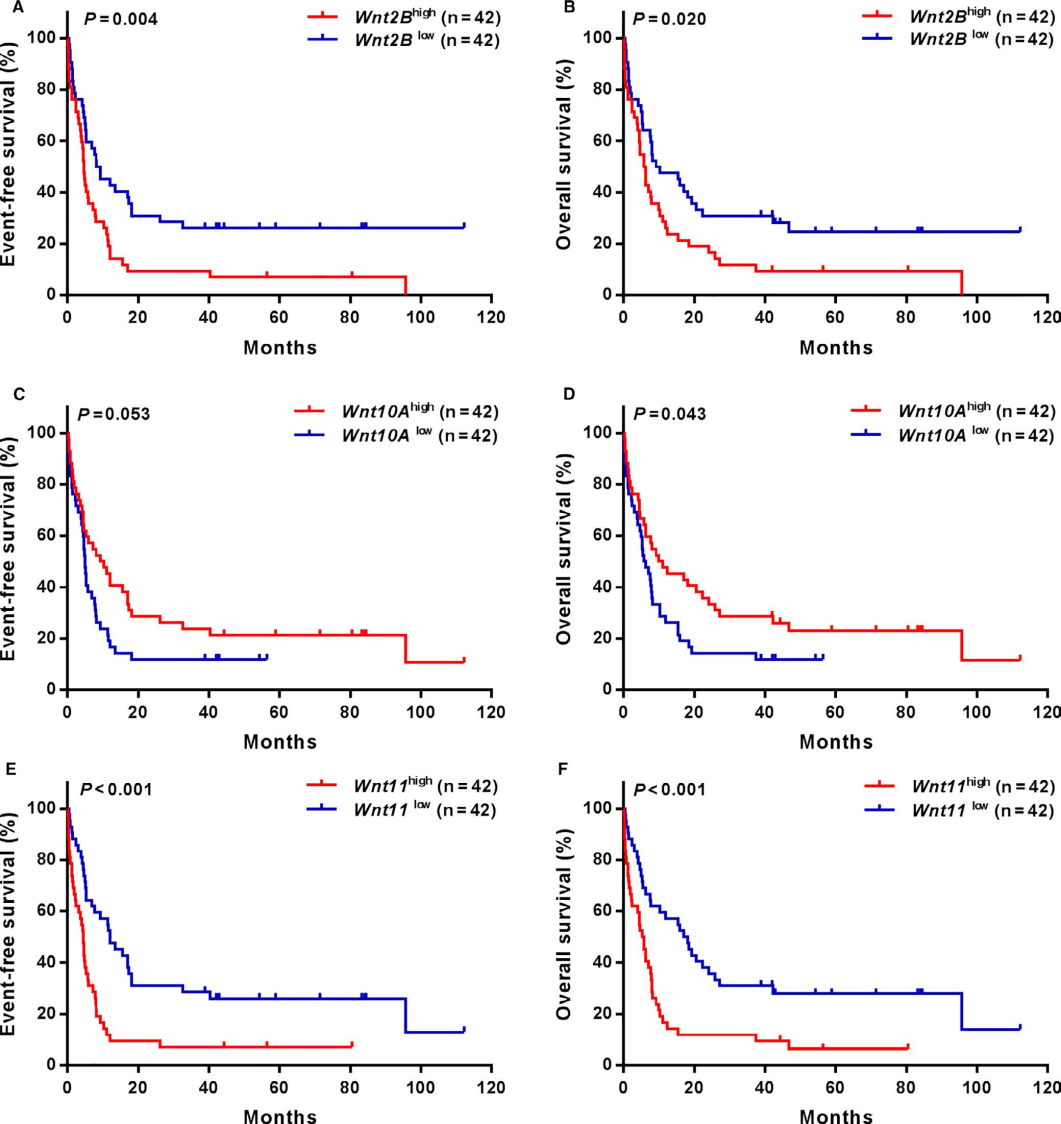
Kaplan‐Meier curves of EFS and OS in patients receiving chemotherapy alone. (A, B) High *Wnt2B* expressers had shorter EFS and OS than the low expressers; (C, D) high *Wnt10A* expressers had longer EFS and OS than the low expressers; (E, F) high *Wnt11* expressers had shorter EFS and OS than the low expressers

According to the median expression levels of Wnt and Fzd family members, AML patients in chemotherapy and allo‐HSCT groups were divided into high and low expression subgroups. Table 1 and Table S1 present the differences of EFS and OS between high and low *Wnt* and *Fzd* gene expression subgroups. The expression levels of all *Fzd* genes were not associated with EFS and OS in both groups except that the expression of *Fzd1* affected EFS in the allo‐HSCT group. All *Wnt* genes were also independent of survival in the allo‐HSCT group (all *P* > .05). In the chemotherapy group, AML patients with high *Wnt2B* expression had significantly shorter EFS and OS compared with low *Wnt2B* expressers (all *P* < .05, Figure 2A,B); high *Wnt11* expressers had significantly shorter EFS and OS than low expressers (all *P* < .001, Figure 2E,F), whereas high *Wnt10A* expression had favourable effect on OS (*P* = .043, Figure 2D).

**TABLE 1:** Comparison of EFS and OS between high and low expression of the *Wnt* gene family

Variables	EFS		OS	
	χ^2^	*P*‐value	χ^2^	*P*‐value
Chemotherapy only group				
*Wnt1* (high vs. low)	3.162	0.075	2.913	0.088
*Wnt2B* (high vs. low)	8.253	0.004	5.454	0.020
*Wnt4* (high vs. low)	0.129	0.719	0.237	0.626
*Wnt5B* (high vs. low)	0.709	0.400	0.552	0.458
*Wnt6* (high vs. low)	2.277	0.131	1.722	0.189
*Wnt8B* (high vs. low)	0.395	0.529	0.085	0.771
*Wnt10A* (high vs. low)	3.754	0.053	4.089	0.043
*Wnt10B* (high vs. low)	0.985	0.321	1.261	0.262
*Wnt11* (high vs. low)	14.055	<0.001	12.442	<0.001
*Wnt16* (high vs. low)	0.254	0.615	0.269	0.604
Allo‐HSCT group				
*Wnt1* (high vs. low)	1.181	0.277	1.726	0.189
*Wnt2B* (high vs. low)	0.091	0.762	0.302	0.582
*Wnt4* (high vs. low)	1.142	0.285	0.088	0.767
*Wnt5A* (high vs. low)	0.214	0.643	2.165	0.141
*Wnt6* (high vs. low)	1.156	0.282	2.260	0.133
*Wnt8B* (high vs. low)	0.321	0.571	0.117	0.732
*Wnt10A* (high vs. low)	0.100	0.752	0.046	0.830
*Wnt10B* (high vs. low)	0.011	0.918	0.672	0.412
*Wnt11* (high vs. low)	0.102	0.750	0.121	0.728
*Wnt16* (high vs. low)	0.429	0.513	0.112	0.738

Abbreviations: Allo‐HSCT, allogeneic haematopoietic stem cell transplantation; EFS, event‐free survival; OS, overall survival.

The verified results showed that AML patients with high *Fzd3* or *Fzd5* expression had significantly longer OS; high expression of *Wnt4*, *Wnt6* and *Wnt7B* also had positive effects on OS, while AML patients with high *Wnt2B* or *Wnt16* expression had significantly shorter OS (all *P* < .05, Figure S1 and Figure S2).

### 3.3 Association of *Wnt2B*/*10A*/*11* expression with clinical and molecular characteristics in the chemotherapy group

As shown in Table 2, *Wnt2B* ^high^ group had fewer AML‐M4 patients than the *Wnt2B* ^low^ group (*P* = .040). No significant differences were found in age, gender, race, WBC, BM blasts, PB blasts, other FAB subtypes, karyotype, risk stratification, relapse ratio and frequency of recurrent genetic mutations (*FLT3*, *NPM1*, *DNMT3A*, *IDH1/IDH2*, *RUNX1*, *NRAS/KRAS*, *TET2*, *TP53* and *MLL*) between the *Wnt2B* ^low^ and *Wnt2B* ^high^ groups (all *P* > .05).

**TABLE 2:** Comparison of the clinical and molecular characteristics between high and low *Wnt2B/10A/11* expression subgroups in the chemotherapy group

Characteristics	*Wnt2B*			*Wnt10A*			*Wnt11*		
	High (n = 42)	Low (n = 42)	*P*‐value	High (n = 42)	Low (n = 42)	*P*‐value	High (n = 42)	Low (n = 42)	*P*‐value
Age/years, median (range)	66.5 (34.0, 88.0)	66.5 (22.0, 82.0)	0.465	67.0 (35.0, 88.0)	65.0 (22.0, 82.0)	0.342	69.0 (34.0, 82.0)	64.0 (22.0, 88.0)	0.09
Age group/n (%)									
< 60 years	13 (31.0)	13 (31.0)	1.000	12 (28.6)	14 (33.3)	0.637	10 (23.8)	16 (38.1)	0.157
≥ 60 years	29 (69.0)	29 (69.0)		30 (71.4)	28 (66.7)		32 (76.2)	26 (61.9)	
Gender/n (%)									
Male	22 (52.4)	23 (54.8)	0.827	20 (47.6)	25 (59.5)	0.274	25 (59.5)	20 (47.6)	0.274
Female	20 (47.6)	19 (45.2)		22 (52.4)	17 (40.5)		17 (40.5)	22 (52.4)	
Race/n (%)									
Caucasian	28 (66.7)	35 (83.3)	0.078	33 (78.6)	30 (71.4)	0.450	30 (71.4)	33 (78.6)	0.450
Others	14 (33.3)	7 (16.7)		9 (21.4)	12 (28.6)		12 (28.6)	9 (21.4)	
WBC/×10^9^/L, median (range)	14.4 (1.7, 297.4)	15.1 (0.7, 116.2)	0.181	8.6 (0.7, 131.5)	41.6 (1.0, 297.4)	0.006	13.3 (1.4, 297.4)	16.1 (0.7, 131.5)	0.993
BM blasts/%, median (range)	73.5 (32.0, 99.0)	70.0 (30.0, 98.0)	0.543	63.5 (30.0, 91.0)	80.5 (40.0, 99.0)	<0.001	71.5 (32.0, 99.0)	72.0 (30.0, 95.0)	0.900
PB blasts/%, median (range)	23.5 (0.0, 98.0)	23.5 (0.0, 97.0)	0.504	11.5 (0.0, 97.0)	51.5 (0.0, 98.0)	0.001	24.5 (0.0, 98.0)	22.0 (0.0, 97.0)	0.882
FAB subtypes/n (%)									
M0	5 (11.9)	2 (4.8)	0.433	1 (2.4)	6 (14.3)	0.109	4 (9.5)	3 (7.1)	1.000
M1	10 (23.8)	10 (23.8)	1.000	8 (19.0)	12 (28.6)	0.306	10 (23.8)	10 (23.8)	1.000
M2	10 (23.8)	11 (26.2)	0.801	9 (21.4)	12 (28.6)	0.450	10 (23.8)	11 (26.2)	0.801
M4	6 (14.3)	14 (33.3)	0.040	14 (33.3)	6 (14.3)	0.040	8 (19.0)	12 (28.6)	0.306
M5	7 (16.7)	5 (11.9)	0.533	7 (16.7)	5 (11.9)	0.533	6 (14.3)	6 (14.3)	1.000
M6	1 (2.4)	0 (0.0)	1.000	1 (2.4)	0 (0.0)	1.000	1 (2.4)	0 (0.0)	1.000
M7	3 (7.1)	0 (0.0)	0.241	2 (4.8)	1 (2.4)	1.000	3 (7.1)	0 (0.0)	0.241
Cytogenetics/n (%)									
Normal	19 (45.2)	21 (50.0)	0.662	21 (50.0)	19 (54.8)	0.662	16 (38.1)	24 (57.1)	0.081
t(9;22)/*BCR‐ABL1*	1 (2.4)	0 (0.0)	1.000	0 (0.0)	1 (2.4)	1.000	1 (2.4)	0 (0.0)	1.000
inv(16)/*CBFβ‐MYH11*	2 (4.8)	4 (9.5)	0.676	5 (11.9)	1 (2.4)	0.202	3 (7.1)	3 (7.1)	1.000
Complex	35 (83.3)	38 (90.5)	0.332	34 (81.0)	39 (92.9)	0.106	33 (78.6)	40 (95.2)	0.024
11q23/*MLL*	2 (4.8)	1 (2.4)	1.000	1 (2.4)	2 (4.8)	1.000	1 (2.4)	2 (4.8)	1.000
‐7/7q‐	3 (7.1)	0 (0.0)	0.241	1 (2.4)	2 (4.8)	1.000	3 (7.1)	0 (0.0)	0.241
t(8;21)/*RUNX1‐RUNX1T1*	2 (4.8)	4 (9.5)	0.676	1 (2.4)	5 (11.9)	0.202	2 (4.8)	4 (9.5)	0.676
Others	6 (14.3)	8 (19.0)	0.558	5 (11.9)	9 (21.4)	0.242	7 (16.7)	7 (16.7)	1.000
Risk/n (%)									
Good	4 (9.5)	8 (19.0)	0.212	6 (14.3)	6 (14.3)	1.000	5 (11.9)	7 (16.7)	0.533
Intermediate	22 (52.4)	24 (57.1)	0.661	25 (59.5)	21 (50.0)	0.381	18 (42.9)	28 (66.7)	0.028
Poor	14 (33.3)	9 (21.4)	0.221	11 (26.2)	12 (28.6)	0.807	16 (38.1)	7 (16.7)	0.028
*FLT3*/n (%)									
*FLT3*‐ITD	10 (23.8)	5 (11.9)	0.350	7 (16.7)	8 (19.0)	0.100	8 (19.1)	7 (16.7)	0.872
*FLT3*‐TKD	3 (7.1)	4 (9.5)		1 (2.3)	6 (14.3)		4 (9.5)	3 (7.1)	
Wild type	29 (69.0)	33 (78.6)		34 (81.0)	28 (66.7)		30 (71.4)	32 (76.2)	
*NPM1*/n (%)									
Mutation	16 (38.1)	11 (26.2)	0.243	11 (26.2)	16 (38.1)	0.243	13 (31.0)	14 (33.3)	0.815
Wild type	26 (61.9)	31 (73.8)		31 (73.8)	26 (61.9)		29 (69.0)	28 (66.7)	
*DNMT3A*/n (%)									
Mutation	12 (28.6)	11 (26.2)	0.807	10 (23.8)	13 (31.0)	0.463	11 (26.2)	12 (28.6)	0.807
Wild type	30 (71.4)	31 (73.8)		32 (76.2)	29 (69.0)		31 (73.8)	30 (71.4)	
*IDH1*/*IDH2*/n (%)									
Mutation	5 (11.9)	10 (23.8)	0.154	7 (16.7)	8 (19.0)	0.776	4 (9.5)	11 (26.2)	0.046
Wild type	37 (88.1)	32 (76.2)		35 (83.3)	34 (81.0)		38 (90.5)	31 (73.8)	
*RUNX1*/n (%)									
Mutation	7 (16.7)	7 (16.7)	1.000	9 (21.4)	5 (11.9)	0.242	6 (14.3)	6 (19.0)	0.558
Wild type	35 (83.3)	35 (83.3)		33 (78.6)	37 (88.1)		36 (85.7)	34 (81.0)	
*NRAS/KRAS*/n (%)									
Mutation	5 (11.9)	7 (16.7)	0.533	7 (16.7)	5 (11.9)	0.533	7 (16.7)	5 (11.9)	0.533
Wild type	37 (88.1)	35 (83.3)		35 (83.3)	37 (88.1)		35 (83.3)	37 (88.1)	
*TET2*/n (%)									
Mutation	4 (9.5)	7 (16.7)	0.332	6 (14.3)	5 (11.9)	0.746	6 (14.3)	5 (11.9)	0.746
Wild type	38 (90.5)	35 (83.3)		36 (85.7)	37 (88.1)		36 (85.7)	37 (88.1)	
*TP53*/n (%)									
Mutation	7 (16.7)	4 (9.5)	0.332	8 (19.0)	3 (7.1)	0.106	9 (21.4)	2 (4.8)	0.024
Wild type	35 (83.3)	38 (90.5)		34 (81.0)	39 (92.9)		33 (78.6)	40 (95.2)	
*MLL*									
Positive	4 (9.5)	7 (16.7)	0.332	6 (14.3)	5 (11.9)	0.746	5 (11.9)	6 (14.3)	0.746
Negative	38 (90.5)	35 (83.3)		36 (85.7)	37 (88.1)		37 (88.1)	36 (85.7)	
Relapse/n (%)									
Yes	18 (42.9)	13 (31.0)	0.258	15 (35.7)	16 (38.1)	0.821	14 (33.3)	17 (40.5)	0.498
No	24 (57.1)	29 (69.0)		27 (64.3)	26 (61.9)		28 (66.7)	25 (59.5)	

Abbreviations: BM, bone marrow; FAB, French‐American‐British; PB, peripheral blood; WBC, white blood cell.

*Wnt10A* ^high^ group had lower WBC (*P* = .006), BM blasts (*P* < .001) and PB blasts (*P* = .001) and more FAB‐M4 patients (*P* = .040) than the *Wnt10A* ^low^ group. Other clinical and molecular characteristics including age, gender, race, FAB subtypes (M0, M1, M2, M5, M6, and M7), karyotype, risk stratification, relapse ratio and frequency of recurrent genetic mutations (*FLT3*, *NPM1*, *DNMT3A*, *IDH1/IDH2*, *RUNX1*, *NRAS/KRAS*, *TET2*, *TP53* and *MLL*) showed no significant differences between the two groups (all *P* > .05).

Compared with *Wnt11* ^low^ group, *Wnt11* ^high^ group had fewer patients with complex karyotype (*P* = .024), fewer intermediate (*P* = .028) and more poor risk patients (*P* = .028), and more frequent *IDH1/IDH2* (*P* = .046) and fewer *TP53* (*P* = .024) mutations. No significant differences were observed in age, gender, race, WBC, BM blasts, PB blasts, FAB subtypes, relapse ratio and other frequency of recurrent genetic mutations (*FLT3*, *NPM1*, *DNMT3A*, *RUNX1*, *NRAS/KRAS*, *TET2* and *MLL*) (all *P* > .05).

### 3.4 Multivariate analysis of possible prognostic factors in the chemotherapy group

In order to further evaluate independent prognostic value of *Wnt2B*/*10A*/*11* in patients treated with chemotherapy only, expression levels of *Wnt2B*/*10A*/*11* (high vs. low), age (≥60 vs. <60 years), WBC (≥15 vs. <15 × 10^9^/L), BM blasts (≥70 vs. <70%), PB blasts (≥20 vs. <20%), *FLT3‐ITD* (positive vs. negative) and some genetic mutations (*NPM1*, *DNMT3A*, *IDH1/IDH2*, *RUNX1*, *NRAS/KRAS*, *TET2*, *TP53* and *MLL*; mutated vs. wild) were included in multivariate analysis. As presented in Table 3, high *Wnt11* expression was an independent risk factor for EFS and OS, along with age ≥ 60, BM blasts ≥ 70% and mutations in *DNMT3A*, *RUNX1*, *TP53* and *MLL* (all *P* < .05); high *Wnt2B* expression and WBC count ≥ 15×10^9^/L were independent risk factors for EFS (all *P* < .05), whereas high *Wnt10A* expression was an independent favourable factor for EFS (HR = 0.0560, *P* = .038) and OS (HR = 0.514, *P* = .018).

**TABLE 3:** Multivariate analysis of the potential prognostic factors of EFS and OS in the chemotherapy group

Variables	EFS		OS	
	HR (95%CI)	*P*‐value	HR (95%CI)	*P*‐value
*Wnt2B* (high vs. low)	2.100 (1.214, 3.632)	0.008		NS
*Wnt10A* (high vs. low)	0.560 (0.323, 0.969)	0.038	0.514 (0.296, 0.893)	0.018
*Wnt11* (high vs. low)	2.550 (1.421, 4.574)	0.002	2.750 (1.544, 4.897)	0.001
Age (≥60 vs. <60 years)	3.024 (1.584, 5.773)	0.001	2.392 (1.253, 4.569)	0.008
WBC (≥15 vs. <15 × 10 /L)	1.823 (1.033, 3.217)	0.038		NS
BM blasts (≥70 vs. <70%)	2.429 (1.373, 4.296)	0.002	2.033 (1.159, 3.565)	0.013
PB blasts (≥20 vs. <20%)		NS		NS
*FLT3‐ITD* (positive vs. negative)		NS		NS
*NPM1* (mutated vs. wild)		NS		NS
*DNMT3A* (mutated vs. wild)	2.486 (1.372, 4.505)	0.003	2.487 (1.384, 4.469)	0.002
*IDH1*/*IDH2* (mutated vs. wild)		NS		NS
*RUNX1* (mutated vs. wild)	3.505 (1.651, 7.438)	0.001	3.292 (1.584, 6.842)	0.001
*NRAS/KRAS* (mutated vs. wild)		NS		NS
*TET2* (mutated vs. wild)		NS	0.536 (0.246, 1.170)	0.118
*TP53* (mutated vs. wild)	3.136 (1.370, 7.179)	0.007	2.497 (1.139, 5.473)	0.022
*MLL* (mutated vs. wild)	2.909 (1.325, 6.387)	0.008	2.920 (1.305, 6.535)	0.009

Abbreviations: BM, bone marrow; CI, confidence interval; EFS, event‐free survival; HR, hazard ratio; OS, overall survival; PB, peripheral blood; WBC, white blood cell.

### 3.5 Joint prognostic effects of *Wnt10A* and *Wnt11* on AML

**FIGURE 3:**
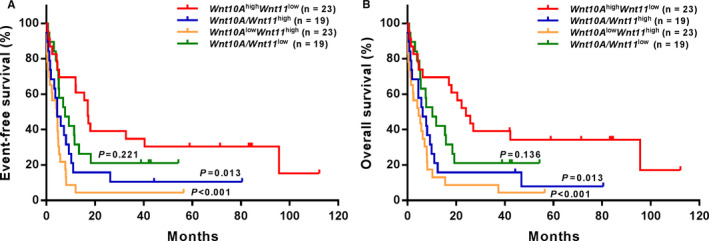
Comparison of EFS and OS between patients with low or high *Wnt10A* and *Wnt11* expression in the chemotherapy group. (A, B) Patients with high *Wnt10A* and low *Wnt11* expression had longer EFS and OS than those with high *Wnt10A* and *Wnt11* expression and those with low *Wnt10A* and high *Wnt11* expression

On the basis of multivariate analysis, we analysed the combined effects of *Wnt10A* and *Wnt11* on prognosis of AML patients receiving chemotherapy only (Figure 3). We found that patients with high *Wnt10A* and low *Wnt11* expression had significantly longer EFS and OS than those in *Wnt10A*/*Wnt11* ^high^ (EFS: *P* = .013, OS: *P* = .013) and *Wnt10A* ^low^/*Wnt11* ^high^ (EFS: *P* < .001, OS: *P* < .013) subgroups. No significant differences of EFS and OS were noticed between the *Wnt10A* ^high^/*Wnt11* ^low^ and *Wnt10A*/*Wnt11* ^low^ subgroups.

### 3.6 Potential mechanism of *Wnt2B*/*10A*/*11* in AML

**FIGURE 4:**
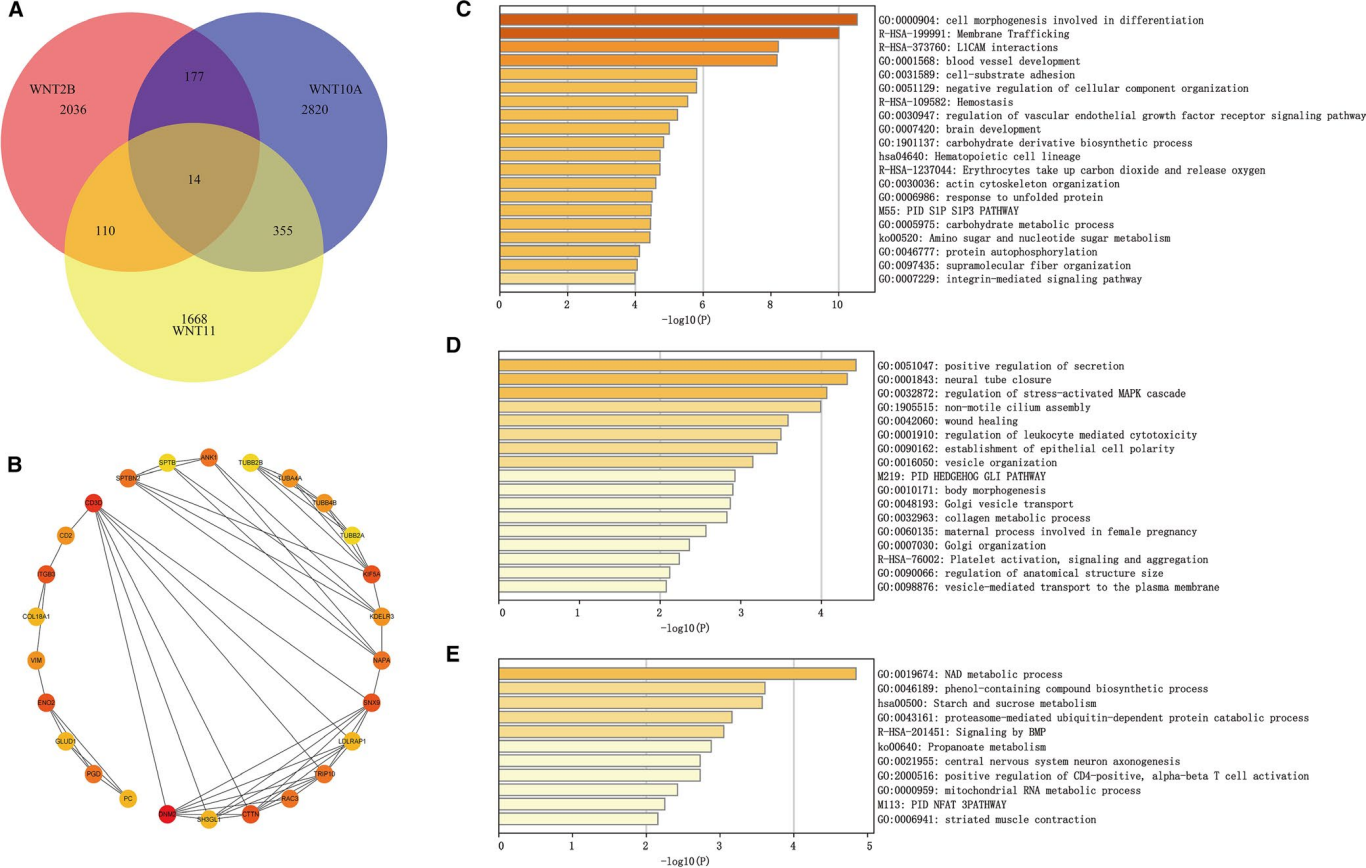
Screening of genes related to *Wnt2B/10A/11* and pathway enrichment analysis. (A) Genes related to *Wnt2B*, *Wnt10A* and *Wnt11* and their intersections; (B) Interaction network of proteins encoded by hub genes; (C, D, E) Pathway enrichment analysis of genes related to *Wnt2B*, *Wnt10A* and *Wnt11*

Our further analysis indicated that the expression of *Wnt2B*, *Wnt10A* and *Wnt11* were significantly correlated with 2337 genes, 3366 genes and 2147 genes, respectively (Figure 4A). Among the intersections of three related gene groups, the number of genes related to both *Wnt10A* and *Wnt11* was the largest (369 genes), and the proteins encoded by these genes had a complex interaction network. The interaction network of proteins encoded by hub genes (top 26; CD3D, SPTBN2, SPTB, ANK1, et al) is shown in Figure 4B. Pathway enrichment analysis showed that genes related to *Wnt10A* and *Wnt11* were mainly enriched in ‘cell morphogenesis involved in differentiation’, ‘blood vessel development’, ‘haemostasis’ and ‘haematopoietic cell lineage’ signalling pathways (Figure 4C); genes related to *Wnt2B* and *Wnt11* were mainly enriched in ‘positive regulation of secretion’, ‘regulation of leukocyte‐mediated cytotoxicity’, ‘Golgi vesicle transport’ and ‘platelet activation, signalling and aggregation’ signalling pathways (Figure 4D); genes related to *Wnt2B* and *Wnt10* were mainly enriched in ‘NAD metabolic process’, ‘proteasome‐mediated ubiquitin‐dependent protein catabolic process’, ‘positive regulation of CD4‐positive, alpha‐beta T‐cell activation’ and ‘mitochondrial RNA metabolic process’ signalling pathways (Figure 4E).

## 4 DISCUSSION

In this retrospective study, we found significant correlations between some members of Wnt and Fzd gene families. The Fzd gene family has no significant prognostic value in AML. High *Wnt2B* and *Wnt11* expression indicates poor outcomes, while high *Wnt10A* expression indicates good outcomes in AML patients with chemotherapy alone, but they have no prognostic significance in AML patients receiving allo‐HSCT.

Nineteen mammalian Wnt ligands can bind to ten Fzd receptors to activate different downstream pathways including Wnt/Ca^2+^, Wnt/β‐catenin and Wnt/planar cell polarity.^19^ Therefore, the correlations between some forms of Wnt and Fzd are reasonable. Down‐regulation of some miRNAs (miRNA‐204, miRNA‐142‐3p, miRNA‐27b) leads to the up‐regulation of *Fzds* in endometrial cancer, cervical cancer and gastric tumour.^20^, ^21^, ^22^ In AML, the knockdown of miRNA‐126 increases the expression of *Fzd7*, leading to aggravation of the disease.^23^ However, we observed that the expression of *Fzds* has no significant effect on the survival of AML patients, which may be because the expression of *Fzds* can be indirectly affected by some miRNAs. The activation of Wnt signalling induces the expression of *ETO* and *AML1* and increases the possibility of their translocation.^24^ AML1‐ETO fusion protein can positively regulate the transcription of plakoglobin, which plays an important role in enhancing the self‐renewal of HSCs.^25^ The expression of *Wnt2B*, *Wnt10A* and *Wnt11* have prognostic value in AML patients receiving chemotherapy only, but not in AML patients receiving allo‐HSCT. The reason may be that allo‐HSCT, as a high‐intensity treatment, temporarily and fundamentally eliminates some translocations, while chemotherapy cannot achieve such an effect.

Wnt signalling pathway has crucial importance in the pathogenesis of AML. There is heterogeneity in Wnt signalling activation in AML, even in groups with the same genotype.^26^ Wnt members, including Wnt3, Wnt4, Wnt5B, Wnt6, Wnt7B, Wnt9A, Wnt10A, Wnt14 and Wnt16, are highly expressed in chronic lymphocytic leukaemia B cells.^27^, ^28^ Nuclear non‐phosphorylated beta‐catenin (NPBC) is produced by activated Wnt signalling pathway, which is associated with poor survival in AML‐M6 and M7 patients.^29^ However, we found that high *Wnt2B* expression subgroup has fewer AML‐M4 patients, whereas high *Wnt10A* expression subgroup has more AML‐M4 patients in the chemotherapy group, suggesting that different *Wnt* genes may be selectively expressed in different AML subtypes. We also found that high *Wnt11* expression is compatible with some poor prognostic factors (poor risk and *IDH1/IDH2* mutations), while high *Wnt10A* expression is incompatible with typical undesirable clinical features (high WBC count, BM and PB blasts) in the chemotherapy group. AML‐associated mutations can activate the downstream Wnt pathway that may contribute to leukaemia pathogenesis.^3^ For example, *NPM1* mutation can activate Wnt signalling by causing the expansion of haematopoietic progenitor cell pool in zebrafish haematopoiesis^30^; *FLT3‐ITD* can activate Wnt/β‐catenin signalling in primary AML cells.^31^ However, we did not find that the expression of *Wnt2B*, *Wnt10A* and *Wnt11* are associated with *NPM1* and *FLT3‐ITD* mutations. The small sample size and the influence of other regulatory factors may be responsible for this phenomenon. This also makes it possible for these three *Wnt* genes to serve as independent prognostic indicators.

Wnt2B is one of the Wnt ligands that stimulate the canonical Wnt pathway.^5^ Wnt2B is associated with clinical stage, T stage and cervical lymph node metastasis in patients with nasopharyngeal carcinoma.^32^ Wang et al reported that silencing *Wnt2B* can decrease the capacity of metastatic dissemination for ovarian cancer cells, and the decreased *Wnt2B* expression can inhibit cancer cell survival and promote cell apoptosis after chemotherapy treatment.^33^ We found that elevated *Wnt2B* expression is associated with worse survival in AML patients with chemotherapy only. This is similar to previous findings that *Wnt2B* expression is significantly higher in human pancreatic cancer tissues than in normal pancreatic tissues, and patients with high *Wnt2B* expression have worse outcomes.^34^ Therefore, *Wnt2B* expression may be used as a new prognostic indicator for AML.

As a member of the canonical Wnt/β‐catenin pathway, *Wnt10A* overexpression is associated with poor survival in patients with idiopathic pulmonary fibrosis.^35^ Li et al observed that overexpression of *Wnt10A* can promote the occurrence and development of human ovarian cancer by activating the Wnt/β‐catenin/TCF/LEF1 signalling pathway. High expression of *Wnt10A* is associated with high tumour grade and advanced stage in patients with ovarian cancer and may be a predictor of patient survival.^36^ *Wnt10A* expression is related to tumour staging and plays a carcinogenic role in the biological process of colorectal cancer.^37^ However, Jiang et al found that the expression of *Wnt10A* in colorectal cancer patients is lower than that in the health group.^38^ We found that high *Wnt10A* expression is associated with good outcomes in AML patients with chemotherapy only. This may be due to the heterogeneity of the expression and function of the same gene in different tumours.

Wnt11 activates both canonical and non‐canonical Wnt signalling pathways and plays controversial role in different cancers. On the one hand, Wnt11, as a tumour promoter, is involved in the proliferation, migration and invasion of various cancers, such as breast cancer, colon cancer, prostate cancer and leukaemia.^39^, ^40^, ^41^, ^42^ On the other hand, Wnt11 is considered to be a tumour‐suppressor protein that inhibits tumour cell migration, invasion and adhesion in ovarian cancer, endometrial cancer and liver cancer.^43^, ^44^, ^45^ We found that high *Wnt11* expression indicates poor outcomes in AML patients with chemotherapy only, suggesting that high *Wnt11* expression may be involved in leukaemogenesis as a tumour promoter.

We found that the prognostic value for *Wnt2B* is still significant, but not for *Wnt11* in the verification cohort lacking *Wnt10A* data. The difference in *Wnt11* results between the validation cohort and our main analysis may be caused by heterogeneity of the two data sets and incomplete information of the validation cohort (no division of chemotherapy and transplantation groups). Moreover, in the subsequent multivariate analysis, *Wnt10A* and *Wnt11* still have significant independent prognostic value even when competing with typical prognostic factors (age ≥ 60 years, BM blasts ≥ 70% and mutations in *DNMT3A*, *RUNX1*, *TP53* and *MLL*) in AML patients with chemotherapy only. Further combined survival analysis also confirmed that AML patients with high *Wnt10A* and low *Wnt11* expression have the longest EFS and OS in the chemotherapy group, suggesting that the combination of *Wnt10A* and *Wnt11* may be a novel prognostic marker for AML and superior to single *Wnt* gene. Pathway enrichment analysis showed that *Wnt10A* and *Wnt11* may participate in the biological process of AML by interacting with genes in ‘cell morphogenesis involved in differentiation’, ‘blood vessel development’, ‘haemostasis’ and ‘haematopoietic cell lineage’ signalling pathways. The specific joint mechanism of them in AML needs to be further clarified.

## CONFLICT OF INTEREST

The authors confirm that there are no conflicts of interest.

## AUTHOR CONTRIBUTIONS

Yifeng Dai and Zhiheng Cheng: collected and analysed the data. Yifeng Dai: drafted the manuscript. Zhiheng Cheng: revised the manuscript. Doerte R. Fricke, Hongyou Zhao, Wenhui Huang, Qingfu Zhong, Pei Zhu, Wenjuan Zhang, Zhihua Wu, Qing Lin, Huoyan Zhu, Yan Liu and Tingting Qian: participate in the data collection and discussion in the revision. Lin Fu, Longzhen Cui and Tiansheng Zeng: conceived and led the study. All authors approved the final manuscript.

## Supporting information

Supplementary Material

## Data Availability

All data sets are available through the Cancer Genome Atlas (TCGA) data portal (https://tcga‐data.nci.nih.gov/tcga). We do not involve direct sample collection. All data analyses of this study have been included in the article.
