# Supplementary material for: Prognostic role of Wnt and Fzd gene families in acute myeloid leukaemia
Source: J Cell Mol Med. 2021 Jan 8;25(3):1456–67. doi: 10.1111/jcmm.16233 (PMC7875934; doi:10.1111/jcmm.16233)
Supplement: Supplementary file 1 — Supplementary Material [file JCMM-25-1456-s001.doc]

**Table S1.** Comparison of EFS and OS between high and low expression of the *Fzd* gene family

| Variables | EFS | |  | OS | |
| --- | --- | --- | --- | --- | --- |
|  | χ^2^ | *P*-value |  | χ^2^ | *P*-value |
| Chemotherapy-only group |  |  |  |  |  |
| *Fzd1* (high vs. low) | 0.001 | 0.981 |  | 0.001 | 0.981 |
| *Fzd2* (high vs. low) | 0.583 | 0.445 |  | 0.511 | 0.475 |
| *Fzd3* (high vs. low) | 0.070 | 0.080 |  | 2.094 | 0.148 |
| *Fzd4* (high vs. low) | 0.439 | 0.570 |  | 0.220 | 0.639 |
| *Fzd5* (high vs. low) | 2.664 | 0.103 |  | 1.577 | 0.209 |
| *Fzd6* (high vs. low) | 2.949 | 0.086 |  | 3.032 | 0.082 |
| *Fzd7* (high vs. low) | 0.278 | 0.598 |  | 0.118 | 0.731 |
| *Fzd8* (high vs. low) | 0.130 | 0.719 |  | 0.487 | 0.485 |
| *Fzd9* (high vs. low) | 0.574 | 0.449 |  | 0.455 | 0.500 |
| Allo-HSCT group |  |  |  |  |  |
| *Fzd1* (high vs. low) | 7.550 | **0.006** |  | 1.582 | 0.208 |
| *Fzd2* (high vs. low) | 0.016 | 0.898 |  | 0.356 | 0.551 |
| *Fzd3* (high vs. low) | 2.269 | 0.132 |  | 0.493 | 0.483 |
| *Fzd4* (high vs. low) | <0.001 | 0.999 |  | 0.209 | 0.648 |
| *Fzd5* (high vs. low) | 0.099 | 0.753 |  | 0.064 | 0.800 |
| *Fzd6* (high vs. low) | 0.353 | 0.552 |  | 1.001 | 0.317 |
| *Fzd7* (high vs. low) | 0.516 | 0.472 |  | 0.574 | 0.449 |
| *Fzd8* (high vs. low) | <0.001 | 0.985 |  | 0.533 | 0.465 |

EFS, event-free survival; OS, overall survival; Allo-HSCT, allogeneic hematopoietic stem cell transplantation.

**Figure legends**

**Figure S1.** Survival curves of Fzd gene family in the verification cohort (GSE12417).

**Figure S2.** Survival curves of Wnt gene family in the verification cohort (GSE12417).
